# Prevalence and risk factors of chronic rhinosinusitis among Chinese: A systematic review and meta-analysis

**DOI:** 10.3389/fpubh.2022.986026

**Published:** 2023-01-09

**Authors:** Lan Zhang, Rong Zhang, Kaiyun Pang, Jie Liao, Chao Liao, Li Tian

**Affiliations:** ^1^Clinical Medical College, Chengdu University of Traditional Chinese Medicine, Chengdu, Sichuan, China; ^2^Department of Otorhinolaryngology, Affiliated Hospital of Chengdu University of Traditional Chinese Medicine, Chengdu, Sichuan, China

**Keywords:** chronic rhinosinusitis, nasal polyps, prevalence, risk factors, Chinese

## Abstract

**Background:**

Chronic rhinosinusitis (CRS) can be seen in people of all ages. CRS heavily affects the quality of a patient's daily life and also causes tremendous economic burdens on patients' families and society. The prevalence of CRS in different countries varies and no systematic review of the prevalence of CRS among Chinese has been published previously. The objective of this systematic review and meta-analysis is to determine the prevalence of CRS among Chinese and to explore the main risk factors of CRS among Chinese.

**Methods:**

Using relevant keywords, data resources including PubMed, Scopus, Web of Science, Google Scholar, Embase, Cochrane Library, Chinese National Knowledge of Infrastructure (CNKI), WANGFANG, VIP, and China Biomedical Literature database (CMB) were searched to obtain literature reporting the prevalence of and risk factors of CRS among Chinese which were clearly diagnosed with CRS from inception to 30 June 2022. The random/fixed effect model was used for meta-analysis, and the I^2^ index was employed to assess heterogeneity among studies. All analyses were performed by using the STATA version 16.0 software. The study was registered with PROSPERO, register number. CRD42022341877.

**Result:**

A total of 12 relevant kinds of literature were qualified for the present systematic review, including 4,033 patients. The results showed that the overall prevalence of CRS among Chinese was 10% (95%CI: 0.06–0.13, I^2^ = 99.6%, *P* < 0.001). The prevalence of CRS among Chinese who lived in urban cities was 18% (95%CI: −0.07 to 0.43, I^2^ = 99.9%, *P* < 0.001), which was obviously lower than the prevalence of CRS among Chinese who lived in rural areas (27%, 95%CI: −0.14 to 0.68, I^2^ = 99.8%, *P* < 0.001). The prevalence of CRS among Chinese before 2010 was 23% (95%CI: −0.05 to 0.50, I^2^ = 99.8%, *P* < 0.001), which was remarkably higher than the prevalence of CRS among Chinese after 2010 (7%, 95%CI: 0.05–0.09, I^2^ = 99.0%, *P* < 0.001). The prevalence of CRS among Chinese who were divorced was 17% (95%CI: 0.12–0.22, I^2^ = 0.0%, *P* = 0.436), while the prevalence of CRS among Chinese who were married, widowed, and unmarried was 9% (95%CI: 0.06–0.11, I^2^ = 88.1%, *P* = 0.004), 9% (95%CI: 0.06–0.11, I^2^ = 0.0%, *P* = 0.863), and 9% (95%CI: 0.08–0.10, I^2^ = 0.0%, *P* = 0.658), respectively. The prevalence of CRS among Han and minority Chinese was 8% (95%CI: 0.07–0.10, I^2^ = 69.6%, *P* = 0.070) and 12% (95%CI: 0.10–0.15, I^2^ = 38.6%, *P* = 0.202), respectively. The prevalence of CRS among Chinese who was never exposed to moldy or damp environments was 8% (95%CI: 0.08–0.09, I^2^ = 0.0%, *P* = 0.351), the prevalence of CRS among Chinese who was occasionally exposed to moldy or damp environments was 16% (95%CI: 0.10–0.22, I^2^ = 78.9%, *P* = 0.030), and the prevalence of CRS among Chinese who was frequently or every day exposed to moldy or damp environments was up to 20% (95%CI: 0.15–0.24, I^2^ = 0.0%, *P* = 0.558).

**Conclusion:**

This meta-analysis shows that the prevalence of CRS among Chinese is at a high level. People who have some risk factors, such as occasional or frequent or everyday exposure to moldy or damp environments, have a higher prevalence of CRS. We should attach more importance to the risk factors of CRS in clinical practice and disseminate scientific information and carry out education to lower the prevalence of CRS in China.

**Systematic review registration:**

https://www.crd.york.ac.uk/PROSPERO/display_record.php?RecordID=341877, identifier: CRD42022341877.

## Introduction

Chronic rhinosinusitis (CRS) is one of the most common inflammatory conditions in otolaryngological diseases and is characterized by chronic inflammation of the paranasal sinus mucosa lasting for >12 consecutive weeks (1). The typical symptoms of CRS are nasal congestion, anterior/posterior rhinorrhea, facial pain or pressure, and reduction or loss of smell. CRS has some considerable influence on the quality of life of patients. Although it is not a life-threatening disease, many patients with CRS cannot be cured or can hardly achieve clinical control, even with a combination of short- or long-course antibiotics, topical, or oral corticosteroids, nasal irrigation with saline, and endoscopic sinus surgery (ESS) (2–5). With greater attention being paid to CRS, increasing studies have been carried out to explore the prevalence, pathogenesis, pathophysiology, and risk factors of CRS.

Increasing epidemiological studies on CRS have been published in Western countries. Trine Thilsing et al. (6) found that the overall prevalence of CRS was 7.8% in a cross-sectional survey of 3,099 subjects in Denmark. In addition, they highlighted that people with occupational exposure to gases, fumes, dust, and smoke or with asthma and nasal allergies have a higher prevalence of CRS (6). According to a survey performed in São Paulo, the prevalence of CRS was 5.51% (7). In a cross-sectional survey of 73,364 Canadians, Chen Yue et al. reported that the prevalence of rhinosinusitis was higher among women (5.7%) than men (3.4%). In addition, the authors highlighted that people who smoked cigarettes and had low income were closely associated with a higher prevalence of rhinosinusitis (8). Klossek et al. reported that the prevalence of nasal polyps (NPs) was 2.11% by performing a cross-sectional, case–control study of 10,033 subjects in France (9). Hastan et al. reported that the overall prevalence of CRS was 10.9% (range 6.9–27.1) among 57,128 subjects aged 15–75 years living in 12 countries in Europe according to E^3^POS criteria (10). Xu et al. reported that the prevalence of CRS in Alberta, Canada, was 18.8 per 1,000 population during 2004–2005, and 23.3 per 1,000 population during 2013–2014 (11). Campion NJ et al. reported that the prevalence of NP was 1.95% in Australia (12). A recent study revealed that the 5-year prevalence of adult CRSwNP cases from 2015 to 2019 in Germany was 374,115 cases (approximately 5,500 per million) (13). Sanchez-Collado et al. reported that the overall prevalence of NP was 0.49% in Catalonia (Spain) and higher for men than women (0.6 vs. 0.39%) (14).

The prevalence of CRS varies in Asian countries. A review revealed that the prevalence of CRS in Asia ranged widely from 2.1 to 28.4% (15). South Korea established the Korea National Health and Nutrition Survey (KNHNS) and published a series of studies about the prevalence and risk factors of CRS in recent years. The prevalence of CRS was 6.95% among 4,098 subjects, and the risk factors for CRS included heavy stress, influenza vaccination, septal deviation, and persistent allergic rhinitis (AR) (16). While in 2008–2012, the prevalence of CRSwNP and CRSsNP in 28,912 adults was 2.6 and 5.8%, respectively (17), in 2010–2012, the prevalence of CRS and CRSwNP among male adults was 3.7 and 0.5% and among female adults 3.3 and 0.3%, respectively (18). In 2008–2012, it was revealed that the prevalence of CRS was substantially higher among 5,590 elderly adults (6.55%) than among 19,939 younger adults (5.69%) according to the EPOS 2012 guideline criteria (19). Kim JH et al. reported that the overall prevalence of CRS based on symptom was as higher as 10.78% (797/7,394), whereas the overall prevalence of CRS based on endoscopy was only 1.20% (88/7,343) (20).

Meanwhile, accumulative studies indicated that the inflammatory patterns of CRS vary in different countries or regions. Notably, 80% of NPs mainly present a distinctive type 2 inflammatory reaction which is marked with infiltration of eosinophils in Europe and North America, while a mixed Th1/Th2/Th17 inflammatory pattern which is predominantly characterized by non-eosinophilic infiltration would always be observed among patients with CRS in East Asian countries, especially in China, South Korea, Japan, and Malaysia. In contrast, the eosinophilic phenotype is less than 50% of CRS cases (21–28). Second-generation Asian patients with CRS also showed a higher prevalence of non-eosinophilic infiltration (29). Moreover, studies revealed that more patients with CRS are likely to exhibit inflammatory patterns other than Th1, Th2, and Th17 in China (30–34).

With the inconstant proceeding of industrialization and urbanization in China, many epidemiological studies on CRS revealed that the prevalence of CRS is constantly rising in recent decades. We noticed that there has been a trend toward increasing eosinophilic NPs among Chinese as well. Understanding the endotypes of CRS helps adopt an optimal and personalized treatment approach, which can not only achieve precision medicine but also can become a breakthrough in preventing and treating CRS (35–38). It is noticeable that numerous risk factors (e.g., genetics/heredity and environmental exposure) contributed to the diverse endotypes of CRS. We found that there was no related meta-analysis study earlier on the prevalence and risk factors of CRS among Chinese by thoroughly searching Chinese and English databases. So, it is meaningful to perform this study to explore the prevalence and risk factors for CRS among Chinese.

## Methods

### Search strategy

The following databases (PubMed, Scopus, Web of Science, Google Scholar, Embase, The Cochrane Library, CNKI, WANGFANG, VIP, and CMB) were searched systematically for articles by using valid keywords that were extracted from terms in related articles and medical subject headings (MeSH). A search strategy was designed for each database using keywords including “sinusitis/epidemiology”[Mesh], “nasal polyps/epidemiology”[Mesh], “chronic rhinosinusitis”[All fields], “prevalence”[Mesh], “epidemiology”[All fields], “incidence”[All fields], “Chinese/epidemiology”[Mesh], and “risk factor”[Mesh]. Finally, searches were performed by designing combinations of the keywords to gather the studies published from inception to 30 June 2022.

### Inclusion eligibility criteria

The subjects of the included study were Chinese patients and were clearly diagnosed with CRS. The style of included study is cross-sectional, which was explicitly provided with the total sample size, the number of patients with CRS, the prevalence of CRS and with/without risk factors of CRS, the diagnostic method, and diagnostic criteria of CRS. The included study had a reasonable research design and corrected statistical methods. The language of the included study is limited to English/Chinese.

### Exclusion eligibility criteria

The language of literature that is non-English/non-Chinese will be excluded. The repeated study will be excluded. Reviews, comments, and lectures will be excluded. Literature with incomplete data and literature that did not provide sufficient original data or diagnosis criteria of CRS will be excluded.

### Data extraction

Records obtained after the primary literature search was imported into and managed by the Endnote 20 software. After removing duplicates, two researchers (L ZHANG and R ZHANG) independently reviewed the text based on the inclusion and exclusion criteria. Any discrepancies between the two researchers were resolved by group discussions. Any disagreement will be resolved by discussing with a third party. The elicited data from all papers were used in the current study and were qualified in the form of a checklist. The checklist included information including the first author's name, year of publication, study location, age, number of men/women, diagnosis criteria of CRS, total sample size, number of CRS epidemiological method, and event of CRS.

### Quality appraisal of the included studies

The quality of the included studies was assessed and scored by two reviewers according to the AHRQ's cross-sectional study quality evaluation recommended by the Agency for Healthcare Research and Quality (AHRQ). The checklists of AHRQ are as follows: (1) define the source of information (survey and record review); (2) list inclusion and exclusion criteria for exposed and unexposed subjects (cases and controls) or refer to previous publications; (3) indicate time period used for identifying patients; (4) indicate whether or not subjects were consecutive if not population-based; (5) indicate if evaluators of subjective components of the study were masked to other aspects of the status of the participants; (6) describe any assessment undertaken for quality assurance purposes (e.g., test/retest of primary outcome measurements); (7) explain any patient exclusions from analysis; (8) describe how confounding was assessed and/or controlled; (9) if applicable, explain how missing data were handled in the analysis; (10) summarize patient response rates and completeness of data collection; and (11) clarify what follow-up, if any, was expected and the percentage of patients for which incomplete data or follow-up was obtained. The answer “yes” is scored 1 point, and “no” or “not clear” is scored 0 point. All included studies were classified as having “low” (0–3 points), “medium” (4–7 points), or “high” (8–11 points) methodological quality. Discrepancies in the scores of included studies were resolved through discussion to reach a consensus or a third party.

### Statistical analysis

Statistical analyses were conducted using the STATA version 16.0 software. The I^2^ index was used to investigate heterogeneity among the studies. I^2^ values of <25%, 25 to 75%, and >75% indicated a low, medium, and high level of heterogeneity, respectively. If I^2^ values of <50% and *P* > 0.10, the fixed effect model was applied for meta-analysis. If I^2^ values of >50% and *P* < 0.10, the random effect model was applied for meta-analysis. Potential publication bias was evaluated by visually inspecting the funnel plots and quantified by Egger's test and Begg's test. Sensitivity analysis was used to evaluate the stability of the meta-analysis result.

## Results

**Figure 1:**
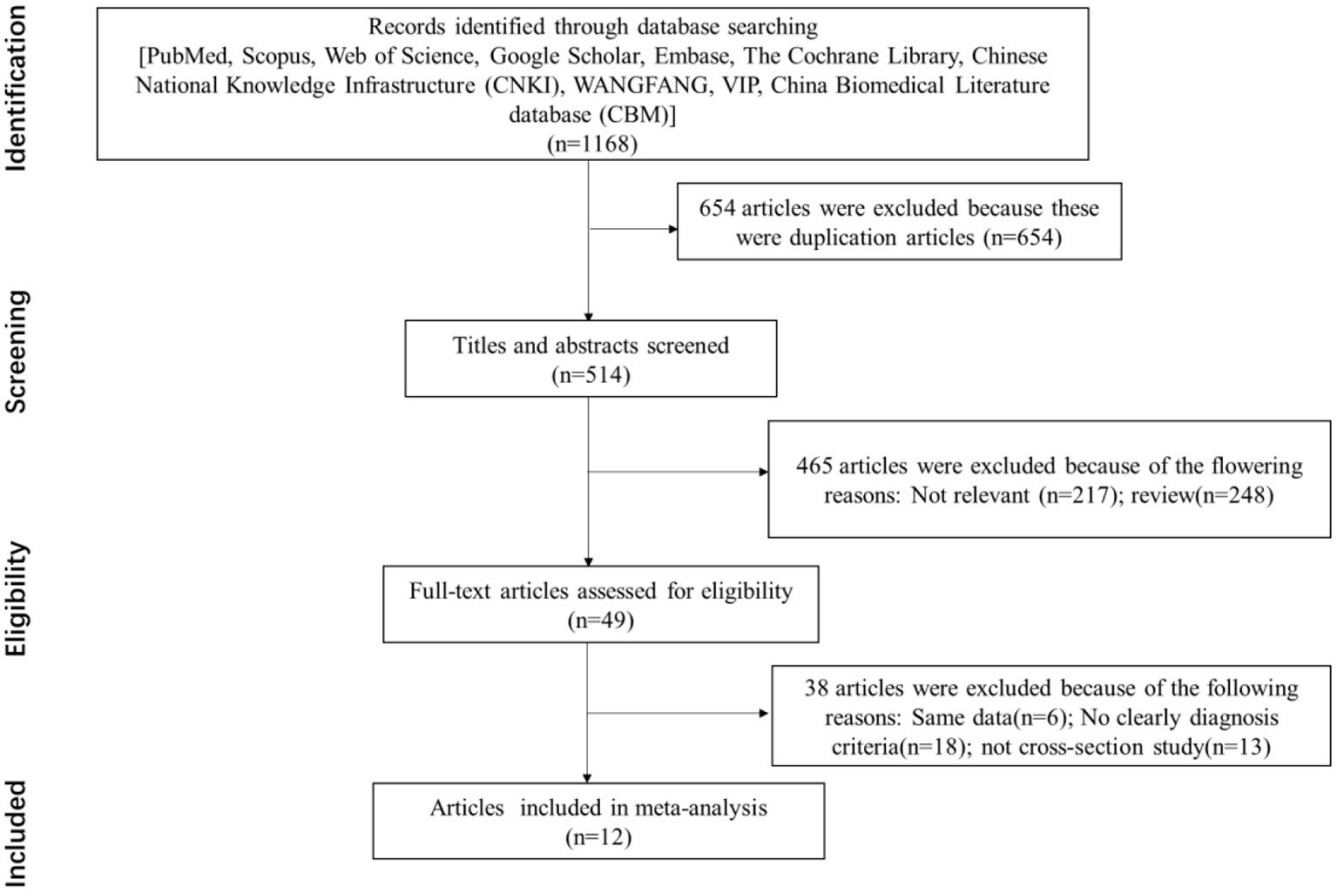
Flowchart of the selection process for eligible studies [the preferred reporting items for systematic reviews and meta-analyses (PRISMA) 2009 flow diagram].

A total of 1,168 studies were identified in the first step of screening. Notably, 654 duplication studies were excluded by reviewing the papers, and 12 studies were qualified for this systematic review eventually (Figure 1). The main characteristics of included studies are summarized in Table 1. Based on the AHRQ checklist, the result of quality assessment in 12 included studies showed that 7 studies had high quality, 5 studies had intermediate quality, and no study had low quality.

**Table 1:** Characteristics of all included studies.

**References**	**Country/** **city**	**Epidemiological method**	**Age**	**Numbers of male/CRS**	**Numbers of female/CRS**	**Diagnosis criteria of CRS**	**Total sample size/numbers of CRS**	**Event of CRS (%)**	**Score**
Shi et al. (39); Gao et al. (40)	China	Stratified four-stage random sampling	>0	5,135/450	5,498/400	A	10,636/851	8.00	8
Fu et al. (41)	China	four-stage random sampling	≥15	689/62	722/56	A	1,411/118	8.36	8
Jiang et al. (42)	Nanjing	Random	9~10	484/44	458/39	B	942/83	8.81	8
Lou et al. (43)	Yiwu	Random sampling	4~14	2,053/742	1,866/697	B	3,919/1,439	36.72	7
Zhao et al. (44)	Changsha	Stratified random sampling	10~17	-	-	C	5,556/280	5.04	8
Zheng et al. (45)	Zhengzhou	Stratified random cluster sampling	-	976/81	946/48	CD	2,020/136	6.73	8
Li et al. (46)	Zhengzhou	Stratified random cluster sampling	-	812/61	818/58	D	1,677/122	7.27	6
Zheng et al. (47)	Jiamusi	Stratified random sampling	-	244/21	210/18	B	454/39	8.59	7
Zheng et al. (48)	Zhengzhou	Stratified random cluster sampling	-	975/-	918/-	D	1,910/119	6.23	7
Gao et al. (49)	Changchun	Stratified random cluster sampling	0~90	739/74	761/74	C	1,500/148	9.87	7
Wang et al. (50)	China	Computerized random digit dialing	16~65	-	-	A	32,931/698	2.12	8

-, not reported; A, European Position Paper on Rhinosinusitis and Nasal polyps (EP^3^OS); B, Complete book of Otolaryngology-Rhinology (second edition) edited by Guo-xuan Bo; C, Chinese guidelines for the diagnosis and treatment of chronic rhinosinusitis (2012, Kunming); D, Chinese guidelines for the diagnosis and treatment of chronic rhinosinusitis (2008, Nanchang). The study conducted by Shi et al. (39) and the study conducted by Gao et al. (40) were derived from the same projects. The projects were from the Industry Foundation of the Ministry of Health of China (201202005), NECT-13-0608, and NSFC (81322012, 81373174, 81170896, 81272062, 81273212, and 81471832).

### The overall prevalence of CRS among Chinese

**Figure 2:**
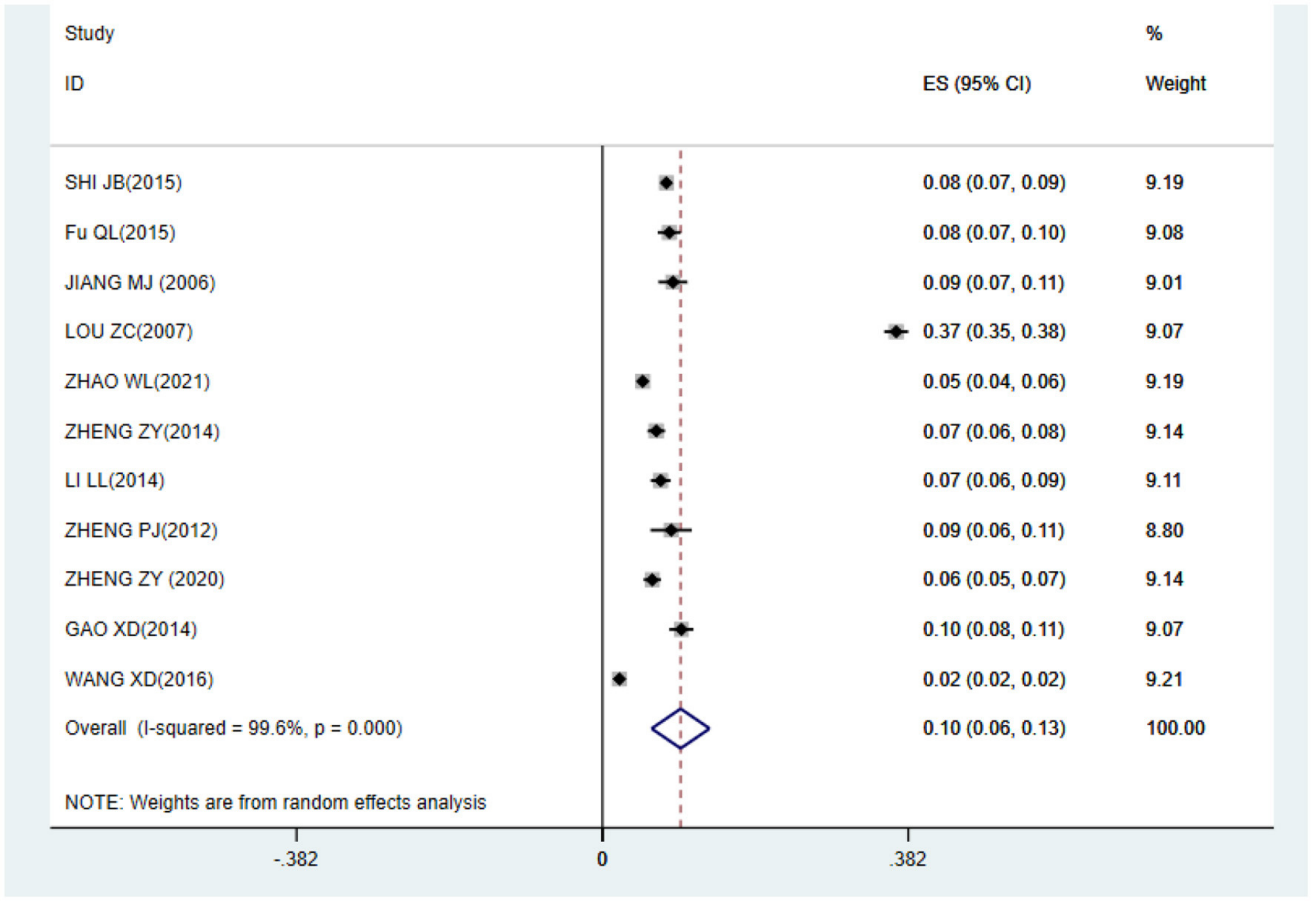
The forest plot of the overall prevalence of CRS among Chinese.

The meta-analysis result shows that the prevalence of CRS among Chinese was 10% (95%CI: 0.06–0.13) (Figure 2).

### Assessment of sensitivity analysis

**Figure 3:**
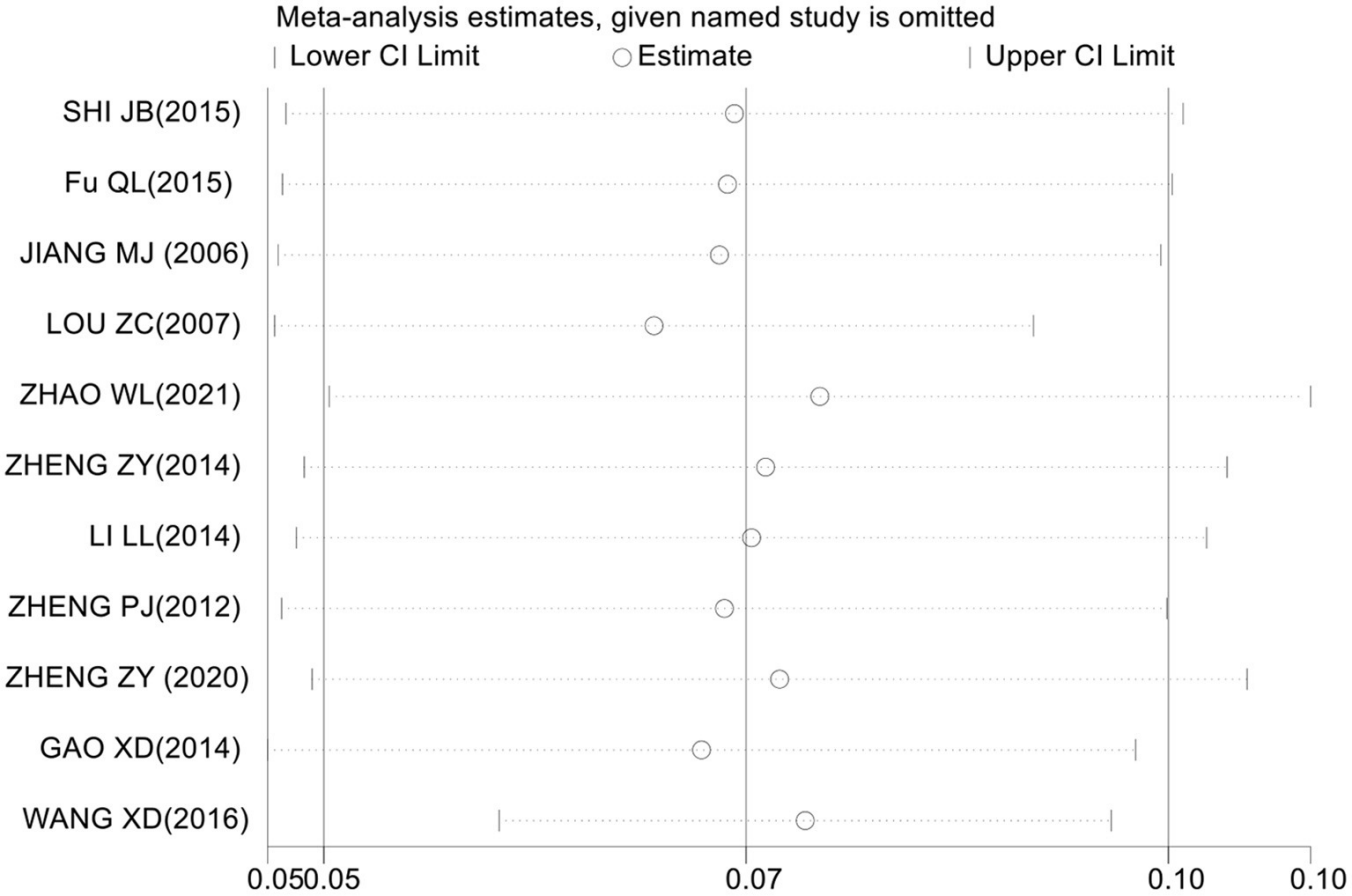
The sensitivity analysis of the overall prevalence of CRS among Chinese.

The assessment of sensitivity analysis results shows that the prevalence of CRS among Chinese did not change significantly compared with the prevalence before omitting an individual study. This result indicates that the results of this meta-analysis are relatively stable (Figure 3).

### Assessment of publication bias

**Figure 4:**
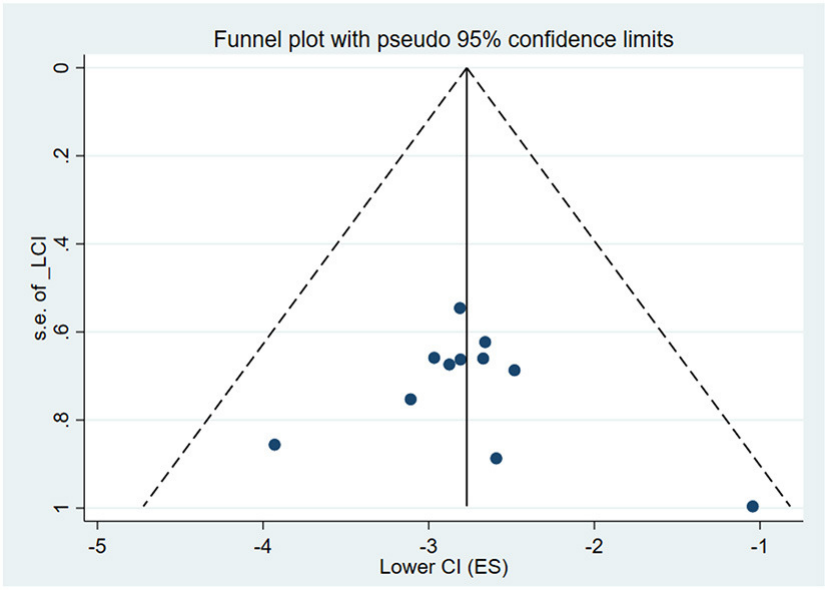
The funnel chart of the overall prevalence of CRS among Chinese.

We performed Begg's and Egger's tests on the ten included studies to assess the publication bias. Begg's test shows Z = 0.93 and *P* = 0.350. Egger's test shows t = 0.04 and *P* = 0.972. The result shows that this meta-analysis had no publication bias. Also, funnel plot test was used to check the meta-analysis publication bias, as shown in Figure 4.

## Discussion

The main purpose of this study was to evaluate the prevalence and risk factors for CRS among Chinese. The results of the present study showed that the prevalence of CRS among Chinese was 10%. In different subgroup analyses, the prevalence of CRS among Chinese who lived in urban cities was 18%, which was significantly lower than the prevalence of CRS among Chinese who lived in rural areas (27%). The prevalence of CRS among people who were divorced was highest (17%). The prevalence of CRS among Han Chinese was 8%, which was lower than the prevalence of 12% among minority Chinese. The prevalence of CRS among Chinese who were never exposed to moldy or damp environments was 8%, while the prevalence of CRS among Chinese who was occasionally exposed to moldy or damp environments is 16%. Most notably, the prevalence of CRS among Chinese who were frequently or everyday exposed to moldy or damp environments was up to 20%. The prevalence of CRS among Chinese who never smoked was 8%, which was obviously lower than the prevalence of CRS among Chinese who smoked (11%) (Table 2).

**Table 2:** Different subgroup analyses in the prevalence of CRS among Chinese.

**Subtype**	**Numbers of studies**	**Heterogeneity assessment**		**Random/fixed effects model**	**Meta-analysis results**	
		**I**^2^ **(%)**	***P***		**Prevalence (%)**	**95%CI**
**Gender**						
Male	8	98.9%	*P < * 0.001	Random	11%	(0.07, 0.16)
Female	8	99.0%	*P < * 0.001	Random	11%	(0.06, 0.15)
**Living location**						
Urban	2	99.9%	*P < * 0.001	Random	18%	(−0.07, 0.43)
Rural	2	99.8%	*P < * 0.001	Random	27%	(−0.14, 0.68)
**Publication year**						
Before 2010	2	99.8%	*P < * 0.001	Random	23%	(−0.05, 0.50)
After 2010	9	99.0%	*P < * 0.001	Random	7%	(0.05, 0.09)
**Marital status**						
Married	2	88.1%	0.004	Random	9%	(0.06, 0.11)
Divorced	2	0.0%	0.436	Fixed	17%	(0.12, 0.22)
Widowed	2	0.0%	0.863	Fixed	9%	(0.06, 0.11)
Unmarried	2	0.0%	0.658	Fixed	9%	(0.08, 0.10)
**Ethnicity**						
Han	2	69.6%	0.070	Random	8%	(0.07, 0.10)
Minority	2	38.6%	0.202	Fixed	12%	(0.10, 0.15)
**Education attainment**						
Illiterate and primary	3	66.2%	0.052	Random	9%	(0.05, 0.13)
Secondary school	3	75.8%	0.016	Random	8%	(0.05, 0.11)
High school	2	0.0%	0.493	Fixed	9%	(0.08, 0.10)
Collage	2	0.0%	0.322	Fixed	8%	(0.08, 0.09)
Masters or above	2	70.5%	0.066	Random	11%	(0.01, 0.20)
**Household monthly income per person**						
< RMB $1,000	3	58.0%	0.093	Random	8%	(0.06, 0.11)
RMB $1,001–3,000	3	61.8%	0.073	Random	9%	(0.08, 0.10)
>RMB $3,000	3	0.0%	0.596	Fixed	7%	(0.06, 0.08)
**Exposure to moldy or damp environments**						
Never	2	0.0%	0.351	Fixed	8%	(0.08, 0.09)
Occasionally	2	78.9%	0.030	Random	16%	(0.10, 0.22)
Frequently or everyday	2	0.0%	0.558	Fixed	20%	(0.15, 0.24)
**Tobacco smoke**						
No	2	75.5%	0.043	Random	8%	(0.06, 0.10)
Yes	2	0.0%	0.678	Fixed	11%	(0.10, 0.12)
		**I**^2^ **(%)**	***P***		**Prevalence (%)**	**95%CI**
**Number of smokers living or working with you**						
0	2	79.9%	0.026	Random	8%	(0.06, 0.10)
1	2	0.0%	0.494	Fixed	9%	(0.08, 0.10)
2	2	0.0%	0.676	Fixed	8%	(0.06, 0.10)
3 or above	2	0.0%	0.906	Fixed	10%	(0.08, 0.13)
**Study location**						
Communities	4	62.3%	0.071	Random	9%	(0.08, 0.10)
Schools	7	99.6%	*P < * 0.001	Random	11%	(0.04, 0.18)

Only one study was performed by using the computer-assisted telephone interview (50). Four studies were performed at subjects' homes from several communities by using a face-to-face questionnaire (39–41, 49), while seven studies were performed at elementary and secondary schools by using a combination of questionnaires and physical examination (42–48). Since the subjects from school were not able to represent well the population, we found that there was a higher prevalence of CRS at schools (11%) than in communities (9%). Although four diagnostic criteria of CRS are adopted, it has the same definition of CRS in three diagnostic criteria except EP^3^OS (a lack of endoscopy or CT scan). The prevalence of CRS among Chinese was not dramatically affected by different diagnostic criteria. Compared to people who lived in urban areas, people who lived in rural areas were suffering from CRS easily owing to terrible living conditions, lower economic statuses, and limited health resources (51–53). Meanwhile, people who lived in remote rural areas were more vulnerable to PM2.5 exposure because of an open fire or a traditional stove for a living (54). It was revealed that the prevalence of CRS before 2010 (23%) was remarkably higher than the prevalence of CRS after 2010 (7%). This result should be interpreted cautiously. First, the result seems to be not conformed to the current prevailing trend of CRS. Second, out of the eleven included studies, only two studies were published before 2010, which may be unable to reflect the actual prevalence of CRS owing to the large heterogeneity between studies. Third, the subjects of the two studies were children and adolescents. Children are vulnerable to suffering frequent viral upper respiratory infections, compared to adults. In addition, as a bacterial reservoir, the role that hypertrophic adenoids played in the development of CRS among children cannot be ignored as well.

According to the results of this study, it is obvious that the prevalence of CRS is closely associated with smoking and exposure to moldy or damp environments. In fact, SHI JB et al. highlighted that people with AR, asthma, chronic obstructive pulmonary disease (COPD), and gout have a higher prevalence of CRS. Gao et al. (40) reported that CRS is strongly associated with occupational and environmental factors, such as cleaning-related jobs, healthcare-related jobs, occupational exposure to dust or poisonous gas, pets at home, large carpets at home or workplace, and exposure to moldy or damp environments. Gao (49) had the same opinion as GAO et al. (40). Zheng et al. (48) highlighted that nasal septum deviation (NSD) is associated with CRS.

Several studies indicated that many factors play a crucial role in the development of CRS. Tint et al. indicated that smoking, allergies, anatomic variations, ciliary dysfunction, asthma, bronchiectasis, and aspirin sensitivity were risk factors and comorbidities associated with CRS (55). Reh et al. (56) reported that several environmental factors may contribute to CRS. Air pollution included carbon monoxide, sulfur dioxide, ozone, nitrogen dioxide (NO_2_), PM_2.5_ (particulate matter 2.5), PM_10_ and tobacco smoke, secondhand smoke (SHS), poison gas, dust, fumes, fiber, mites, and diesel fume exposure (56). Wee et al. highlighted that high concentrations of NO_2_ are related to the development of CRS. The odds ratio for CRS would increase 5.40 times when the NO_2_ level increased by 0.1 ppm (57). Another review conducted by Schwarzbach et al. also indicated that cigarette smoking contributed to CRS, either through active smoking or passive exposure to SHS (58). Ostovar et al. indicated that patients who smoke or who worked as healthcare or cleaner have a higher prevalence of CRS (59). The risk factors for MRI abnormalities that were suspected of sinusitis in a Japanese community-dwelling middle-aged and elderly population are obesity, a smoking habit, and a history of asthma or chronic bronchitis (60). A study from Michigan revealed that exposure to SHS is common and significantly independently associated with CRS. Approximately, 40% of CRS appeared to be attributable to SHS (61). A review showed that there is a strong correlation between active and passive cigarette smoke with the prevalence of CRS (62). The significant association between sinusitis and SHS had been proved by a systematic review (63).

Meanwhile, Reh et al. conducted a case–control study in Washington country and found that exposure to SHS during childhood and adulthood may be a risk factor for CRS. Patients who were exposed to SHS had worse nasal symptoms (64). Clarhed et al. found that occupational exposure to paper dust, cleaning agents, metal dust, animals, and moisture/moldy/mildew was independently related to having CRS (65). A recent review carried out by Leland et al. found that air pollution (particularly PM) was closely correlated with CRS incidence/prevalence and disease severity (66). Additionally, Alkholaiwi et al. highlighted that CRS symptoms' severity increased with direct contact with allergens, and the greatest proportion of patients with CRS was found among those with blue-collar occupations, such as firefighters, farmers, and fishermen (67). Mady et al. indicated that the exposure level of air pollutants significantly correlated with CRS symptom severity, particularly with a more pronounced impact on patients with CRSsNP (68). A cross-sectional study conducted in Sweden by Ahlroth Pind et al. demonstrated an independent association between dampness at home and CRS in adults (69). Those findings are consistent with this study's results. Although nasal mucosa has excellent regeneration potentials, the hazards of occupational and environmental pollutions exposure are mainly attributed to inducing chronic, long-term, local, and systematic inflammation, mediated by multiple pathways such as disrupting nasal mucosa epithelial cell cilia, changing sinus bacterial colonization (e.g., decreasing bacterial microbiome richness and diversity), promoting the formation of bacterial biofilm and reactive oxygen species (ROS), increasing the secretion of pro-inflammation cytokines, and impairing nasal mucociliary clearance and epithelial barrier function as well as immune balance (70–81). Those reactions eventually lead to patients developing CRS.

In summary, many factors play a critical role in diseases of the upper airway, including CRS. With the high prevalence and healthcare costs of CRS, more high-quality population-based epidemiological studies are warranted in the future.

## Conclusion

Generally, the results of this study indicated that the prevalence of CRS among Chinese was 10%. The onset of CRS is closely correlated with some factors, such as exposure to moldy or damp environments and SHS. Considering the prevalence of CRS improving in recent decades, the numerous risk factors of CRS, and the burden of CRS in terms of worsening quality of life, as well as other side effects, more studies are warranted to be conducted with a focus on different risk factors, to provide a great deal of insight into CRS and aid healthcare stakeholders in planning useful strategies to decrease the prevalence of CRS.

### Limitation

There are some limitations associated with this study. First, this meta-analysis was based on a cross-sectional study only. There may be sampling errors in the included studies that affect the accuracy of the meta-analysis results. Second, we did not perform analysis on age subgroups because of a lack of agreement. Therefore, we are not certain about the prevalence of CRS among Chinese in different age subgroups. Third, we excluded articles with incomplete information or that did not have a clear diagnosis criterion of CRS. The sample of included studies was relatively small. This may have an impact on the accuracy of the meta-analysis results. Moreover, the subjects in the seven included studies were from elementary and secondary schools which may lack broad representativeness. Therefore, this study may be unable to reflect the actual prevalence of CRS. Finally, there were numerous risk factors for CRS in the included studies that could not be agreed upon. We are not capable of analyzing all risk factors for CRS. It is necessary to expand the included study types to obtain a more convincing result. Despite these limitations, our study is the first meta-analysis to explore the prevalence and risk factors for CRS among Chinese.

## Data availability statement

The original contributions presented in the study are included in the article/supplementary material, further inquiries can be directed to the corresponding author.

## Author contributions

LZ, RZ, and LT designed this study. KYP, JL, and CL were responsible for the literature search and extraction of the data. LZ and RZ were involved in analyzing the data and drafting the manuscript. All authors have read and approved the final manuscript.
